# Perceptions of Undergraduate Students towards Utilization of Clinical Simulation in Teaching and Learning in Rural Universities in Uganda: A Mixed method study

**DOI:** 10.21203/rs.3.rs-4362808/v1

**Published:** 2024-05-28

**Authors:** Pamella Rosset Adongo, Joshua Epuitai, Paul Oboth, Felister Apili, Edward Kumakech, Samuel Owusu-Sekyere, Julius N. Wandabwa

**Affiliations:** Busitema University; Busitema University; Busitema University; Lira Hospital; Lira Hospital; Kwame Nkrumah University of Science & Technology Kumasi; Busitema University

**Keywords:** Perception, Undergraduate students, simulation, clinical teaching, Uganda, rural Universities

## Abstract

**Background:**

Simulation is a new pedagogical method in Africa that underscores the need to operationalize it and generate context-specific scholarship regarding clinical simulation in Africa. Despite the superior learning outcomes of using simulation in medical education, its use in developing countries is often limited, compounded by limited scholarship on simulation especially relevant to an African context. The research aimed to explore perceptions surrounding the use of simulation among undergraduate students.

**Methods:**

A mixed method convergent parallel design was used in which both the quantitative and qualitative approaches were employed currently to explore the perception of the undergraduate students towards the use of clinical simulation in teaching and learning. The quantitative approach assessed the perception of the students on a five-point Likert from strongly disagree to strongly agree scale while the qualitative approach employed a focused group discussion to explore the perception of the students in regards to clinical simulation. Quantitative data was analyzed using Stata Version 17. Qualitative results were analyzed through thematic analysis by Brauna and Clark.

**Results:**

298 participants were recruited into the study. The mean age of the participants was 27 years with a standard deviation of 5.81 years. The majority 152(51.01%) of the participants were males. Most 111(37.25%) of the participants were pursuing Bachelor of Medicine and Bachelor of Surgery. The grand mean of the perception scores of the students towards the use of simulation in clinical teaching was 3.875269 ±0.5281626. The participants strongly agreed that it is incredibly helpful to have someone who works in the field debrief with them after completing a scenario and their overall perception towards this was high (Mean =4.65241±0.6617337).

**Conclusion:**

This exploratory study revealed that medical, nursing, and midwifery students from the rural Universities of Busitema and Lira demonstrated a positive perception of the use of clinical simulation in teaching and learning. The results conclude that simulations help students better understand concepts in clinical settings, provide them with valuable learning experiences, and help them stimulate critical thinking abilities. Further, the participants perceive simulation to be realistic, and knowledge gained could be transferred to the clinical areas.

## Background

The rising number of health students coupled with advancing technology has necessitated to adoption of new methods in teaching [1]. The traditional apprentice model of medical and nursing training has enormous inherent weaknesses [2]. The apprentice model of, “*see one, do one, and teach one*” causes’ performance anxiety as students get to practice on a real patient for the first time without prior experience [1–3]. The traditional model limits students’ ability to experience and or acquire clinical skills for rare clinical conditions, while a high volume of students relative to the number of patients comprises the quality of the bedside traditional model of clinical teaching [2, 3].

Clinical simulation offers numerous superior benefits compared to the traditional model of teaching [5]. Clinical simulation mimics a real-life clinical setting [6] and offers an opportunity for students to learn, re-learn, and make mistakes while uncompromising patient safety and reducing students’ performance anxiety [3–5, 7]. Besides the acquisition of clinical psychomotor skills and compatibility with ethical principles [4, 5], simulation enables students to acquire cognitive soft skills such as inter-professional collaboration, teamwork, and communication skills among many other skills which ordinarily were not possible in the traditional model of teaching [5, 8].

Although Simulation has been in use since the 16th century and is widely used in developed countries, and its utilization in resource-constrained settings such as Uganda is still low [7, 9]. The associated high cost of equipment for simulation, the lack of infrastructure, and human

 resources have been noted to limit the utilization of simulation in low-income countries [8, 10]. Although Clinical simulation offers students more time to practice and hone their clinical skills in a safe learning environment, the, negative perceptions towards the use of clinical simulation may hinder its utilization in medical training.

A limited number of studies in Uganda have generated context-based evidence vital to scaling up the utilization of clinical simulation among health professionals. The purpose of this study was fill the knowledge gap by exploring the perception of undergraduate students towards utilization of clinical simulation at Busitema University and Lira University in Uganda.

## Materials and methods

### Study design

A mixed method convergent parallel design was used in which both the quantitative and qualitative approaches were employed currently to explore the perception of the undergraduate students towards the use of clinical simulation in teaching and learning.

### Study site

This study was conducted at Busitema University and Lira Faculty of Health Science. Both universities have a skills laboratory, while Busitema University has an additional simulation center that provides students with opportunities to acquire clinical skills. Busitema University had undergraduate nursing, anesthesia, and medicine programs, while Lira University had a Bachelor of Midwifery Sciences. All the program follows a competency-based curriculum and provides graduate nurses, anesthetists, and medical students with opportunities to learn clinical and professional skills through simulation-based learning. The undergraduate programs at Busitema University currently admit about 25–30 nursing students, 20–25 anesthesia students, and 60–70 medical students annually. Lira University admits about 60 midwifery students in a year.

### Study population

The study was conducted among students of nursing, midwifery, and medicine. This will include students in clinical years in Busitema University starting from the third year to the final year of study and students in third to fourth year in Lira University.

### Sample size determination

The combined universities have approximately 300 students in the clinical years. Therefore, the whole population of students was included in the study because of the small population. As such, we invited all members of the study population to participate in the study. The sample size for qualitative was obtained by saturation.

### Eligibility criteria

#### Sampling technique

For quantitative, consecutive sampling was used to select participants for the study while purposive sampling was employed for qualitative.

#### Data collection tools and data collection procedure

A self-administered questionnaire was used to assess the status of the utilization of the simulation. The questionnaire will assess the readiness to conduct simulations including the equipment that is available for simulation activities, the availability of scenarios developed, the number of simulation scenarios conducted in a semester, the number of simulation sessions conducted in the skills laboratory, and the courses for which simulation was conducted. The five-point Likert scale was used to assess the perception of the undergraduate, medical, nursing, and midwifery students toward the use of clinical simulation in clinical teaching.

#### Rigors/ trustworthiness of the study

The trustworthiness of the study was ensured by the following terms: credibility, transferability, conformability, and dependability [3].

##### Credibility

This was ensured through establishing a conducive rapport with participants and conducting a pilot interview before collecting data so as to include questions that might have probably been missed out and also, omitted irrelevant questions. Open-ended questions were used and the participants were given ample time for interaction to allow them express their views fully. The focus group discussions were able to capture all the information from the students without any omission.

##### Conformability

The researcher made vigorous attempts to be objective, which therefore ensured provision of accurate and relevant data. This was done by evaluating interview questions to ensure that they are open ended and not leading questions. Codes used for each participant was used to allow proper matching of each participant’s description.

##### Dependability

Careful listening to the recorded responses to explain phrases that are used by the participants was done. All the activities were recorded from the first step of the study. The research team agreed on the patterns of phrases and categories so that the data remains solid.

##### Transferability

This was achieved by eliciting rich descriptions of study methods and population, adequate sampling and through achieving data saturation point.

### Data management and Analysis

The quantitative approach assessed the perception of the students on a five-point Likert from strongly disagree to strongly agree scale while the qualitative approach employed a focused group discussion to explore the perception of the students in regard to clinical simulation. Quantitative data was analyzed using Stata Version 17 in which descriptive statistic was used to assess the perception of the students.

This for qualitative data, the study employed Braun and Clarke’s six-phase approach to thematic analysis [4] to analyze the data. The phases are “(i) familiarizing the researcher with the data, (ii) generating the initial codes, (iii) searching for themes, (iv) reviewing themes, (v) defining and naming themes, and (vi) producing a report”.

Data were presented narratively. The reporting of data is as per the consolidated criteria for reporting qualitative research (COREQ) guidelines [5].

## Results

### Demographics of the participants

A total of 298 participants were recruited into the study. The mean age of the participants was 26.7 years with standard deviation of 5.81years (Table 1). The majority of the participants 152(51.01%) were males. Most of the participants 111(37.25%) were pursuing Bachelors of Medicine and Bachelors of Surgery.

### Utilization of clinical simulation

The use of clinical simulation was determined using direct questions on how many times students used simulation in an academic semester or indirectly by asking the number of times students spent in the skills or simulation lab. Overall, close to 50% of students had never been to a skills/simulation lab or were there once or a few times which underlines low utilization of clinical simulation (Table 2). The predominant course for simulation was the Obstetrics and gynecology course.

#### Preference of students to learn using clinical simulation.

**Figure 1:**
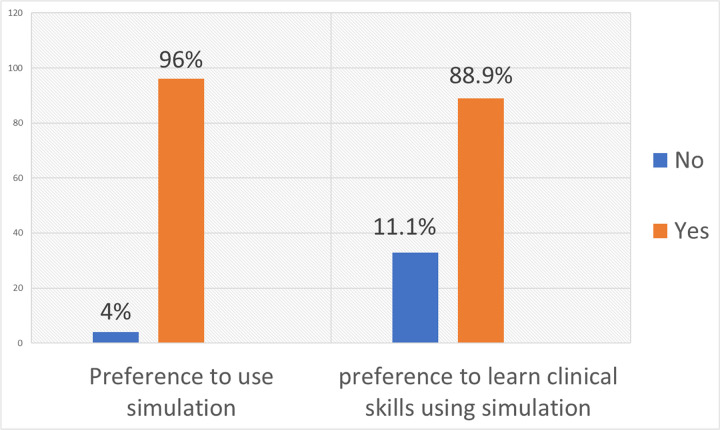
Preference of students to learn using clinical simulation

Preference to use simulation was asked in different ways of which all of them gave a similar response. Although the utilization of clinical simulation was low, 96% of the students preferred to learn clinical skills using clinical simulation (Fig. 1).

### Perception of students towards use of simulation in clinical teaching

The grand mean of the perception scores of the students towards use of simulation in clinical teaching was 3.87 ± 0.528. The participants strongly agreed that it is incredibly helpful to have someone who works in the field debrief with them after completing a scenario and their overall perception towards this was high (Mean = 4.65 ± 0.66). In addition, the participants agreed that it’s important to have simulation experiences because the nurses are not too helpful especially during the first week of clinical placement (Mean = 3.750446 ± 1.081552). The participants disagreed with the thinking that simulation is very stressful and over whelming (Mean = 2.365772 ± 1.204832). Furthermore, the participants were neutral about not really learning many interpersonal skills from practicing on a doll (Mean = 3.218127 ± 1.32678). The results are summarized in Table 3 (Additional File 1)

### Qualitative Results

#### Qualitative results on the Perception of the students towards the use of clinical simulation in teaching

Three themes emerged regarding the satisfaction of the students with the use of clinical simulation in teaching and learning; these include “mismatch between number and students and available space in the simulation laboratory”; Simulation not integrated into the training curriculum; and Low fidelity of the simulation (Table 4).

### Theme one: Mismatch between student numbers and available space in the simulation laboratory

One of the major factors that emerged among the participants is the mismatch between student numbers and available space in the simulation laboratory. The student perceived the number of students to overwhelm the available simulation laboratory:

“…*I am not satisfied. This is because the number of students assigned to a patient are many and it cause collision and there is lack of communication. The ratio of students to patient should be something like 5:1 instead of*……”

[FGD 13b Midwifery].

“…*yes I would say partially it’s fine because this simulation is only in 2 departments, that is, anesthesia and nursing. For the medicine, we do not do simulation. Even with nursing, the space at the simulation center is actually not enough*….”

[FGD 3b]

### Theme two: Simulation not integrated into the training curriculum

The second theme that emerged from the participants is “Simulation not integrated into the training curriculum”. They verbalized this to be one of the major reasons for their dissatisfaction with simulation. This was expressed in terms of simulation not being aligned with theory classes, simulation not being included in the normal teaching timetable but only during examinations:

“…*I am not satisfied with simulations because we only have them when it is a progressive assessment or there’s someone doing a research study and they want to*…..….”

[FGD 14b Midwifery].

“…*I am not satisfied, Some of the barriers, challenges yeah simulation has not been included in the university curriculum and you find that it is only done because there is actually an exam but it is not as part of the day to day lectures students are having*……”

[FGDA1]

### Theme three: Low delity of the simulation

The final theme that emerged from the participants is “Low fidelity of the simulation”. The participants perceived simulation not to be real when compared to clinical practice. They stated this to be one of the major reasons for their dissatisfaction:

“… *I am not satisfied in a way that there is a lot of imagination you will find that you want to administer maybe oxygen they tell you it is already connected like you want to administer any IV fluid you bring a cannular and tie it with a strip so there is a lot of imagination*….”

[FGD 14b Midwifery]

*I find myself being extra confident when I am doing a simulation during simulations because I know at times I am not managing the real life*…..”

[FGD A6 midwifery]

We further explored the perception of the students towards the possibility of translating simulation skills to real practice. Generally, the students perceived the skill acquired in clinical simulation to be translatable to real clinical practice patients care:

“…….*Simulation are actually teaching sessions, so if I get to know something from a simulation and I am faced with it in real life, what hinders me from actually applying it?*…….

[FGD A1]

*“……………I think the skills translate because some things that we have learned from simulation have been able been able to also do them in te clinical setting and they have really helped me……………*.”

[FGD A2].

## Discussions

This study investigated the perception of the undergraduate, medical, nursing and midwifery students towards the used of clinical simulation in clinical teaching.

Generally, the students had good perception towards the use of medical simulation in clinical teaching. This is demonstrated by their grand mean of their perception scores of the Likert scale to be 3.875269 ± .5281626. This grand mean of their perception score is greater than the criterion/Decision mean which “3” for a five-point Likert scale.

This finding of good perception of medical, nursing and midwifery students towards use of clinical simulation in teaching and learning has been reported in other related studies elsewhere [6–14].

Expounding further on this, Aalto, Heponiemi [6], found that overall, about 170(73.9%) of the students were very satisfied with use of clinical simulation in teaching and about 191(83%) of the medical students were satisfied with instructors’ cooperation. Madhavanprabhakaran, Al-Khasawneh [9] in Oman found that undergraduate nursing students perceived simulation to facilitate enhancement of their knowledge and skills in regards to clinical nursing. El Naggar and Almaeen [10] in Saudi Arabia reported that about 170(73.9%) of the students were satisfied with the use of clinical simulation in teaching and learning. Codeço, Dias Coutinho [12], in Japan demonstrated that students perceived clinical simulation to “contributes to an active participation of learners in their learning process, promotes” the development of skills such as communication, priority management and decision-making.

Elliott and Brumbaugh [13] reported that the simulation learning experience led to a positive perception in regards to utilization of simulation in assessments and understanding of the testing procedures. Furthermore findings among nursing students in a large study conducted among students in the United kingdom and Saudi Arabia Universities revealed that students perceived clinical simulation in teaching and learning to enhance their “self-awareness, self-confidence, clinical performance, and efficiency” in practicing the nursing procedures [14], However, the findings are contrary to other studies elsewhere [15, 16]. Nel and Stellenberg [15] found that though the students were comfortable with clinical, they felt more confident and competent after practicing on humans. Woodruff, O’Neill [16] on the contrary found that generally the students were undecided (neutral) in regards to use of clinical simulation to facilitate teaching in the University of California.

The possible explanation for these inconsistences between the findings of the present study and that of other related studies elsewhere could be due to differences in study settings and study designs For example Nel and Stellenberg [15] assessed not only perception of the students in regards to clinical simulation but their study also compared clinical simulation with the real patient practice which our study did not measure. Similarly, Woodruff, O’Neill [16] conducted their study from California and among the advanced practice nursing students of which some of the students had experience with clinical simulation. This is therefore a possible explanation for the inconsistency between the findings of the present study and their study findings as our study was conducted among the undergraduate medical, nursing and midwifery students with no prior exposure to clinical simulation before joining the study Universities.

Findings from this study demonstrated that students strongly agreed with the statement of having someone to having an instructor who works in the field to be present during the debriefing phase of clinical simulation. This finding is consistent with that in related studies elsewhere [6, 11]. Aalto, Heponiemi [6] found that the nursing students acknowledged good cooperation from the simulation instructors from the real filed in the simulation session. García-Mayor, Quemada-González [11], found that students strongly agreed that the presence of qualified healthcare professionals for example qualified nurses enriched the simulation experience. This noted that the medical students agreed to having clinical simulation during the first week of clinical placement.

This findings is similar to that in other related studies [17]. García-Mayor, Quemada-González [17] found that the students perceived simulation to prepare them for clinical years. In regards to facilitating the realization of student’s areas of weakness that need improvement, the students strongly agreed that clinical simulation facilitates the awareness of one’s ability, strengths and weakness which are then improved before interfacing with the actual patients. This findings form this study of clinical simulation being an avenue for skill improvement is consistent with findings from other studies elsewhere [10]. El Naggar and Almaeen [10] found that majority 147(74.2%) of the students reported simulation to help improve on their practical and communication skill.

Though the students had overall positive perception towards the use of clinical simulation in teaching and learning, there satisfaction is still deficient. This was expressed in terms of “mismatch between number and students and available space in the simulation laboratory”; Simulation not integrated into the training curriculum; and Low fidelity of the simulation. These findings have been reported in other related studies [18]. El Naggar and Almaeen [18] found that students expressed their poor satisfaction with simulation in terms of “lack of proper training rooms 40/230(17.40), shortage of training times 80/230(34.50%), inadequate facilities 52/230(22.6%)”.

## Conclusion

This study’s findings support the integration of the simulation in the curriculum, as evidenced by the positive perception of the medical, nursing and midwifery students at the rural universities of Busitema and Lira. Simulations help students better understand concepts in clinical settings, provide them with valuable learning experiences, and help them to stimulate critical thinking abilities. The participants perceived simulation sessions to be realistic, and that the knowledge gained from simulations could be transferred to the clinical areas. Therefore there is need to escalate the use of simulation as an instrument of teaching and learning with the need for more investment in terms of infrastructure and equipment which will make the simulation more real and closer to the real world that students experience in the wards. Furthermore, there is a need for curriculum revision to include simulation within the existing curriculum.

## Figures and Tables

**Table 1:** Socio-Demographic Characteristics of the participants

Variable	Frequency (%)
Gender	
Male	152(51.0)
Female	146(49.0)
Institution	109(36.6)
Lira University	189(63.4)
Busitema University	
Course	
Anesthesia	40(13.4)
Medicine	111(37.2)
Midwifery	109(36.6)
Nursing	38(12.7)
Mode of entry	
Direct	208(69.8)
Extension	90(30.2)
Year of Study	
Year 3	106(35.6)
Year 4	139(46.6)
Year5	53(17.8)

**Table 2:** Utilization of clinical simulation in Lira and Busitema University

Variable	Frequency (percentage)
No. of times been in the skills Lab/Simulation lab	
Most times or frequently	147(49.3)
One or a few times	142(47.7)
Never	09(3.0)
Courses covered in simulation	
Anesthesia and critical care	37(12.4)
Basic Life support	10(3.3)
Medical Nursing/Internal Medicine	46(17.1)
Obstetrics/Gynecology/Midwifery/Neonate	153(57.1)
Pediatric Nursing	13(4.4)
Surgical nursing/Surgery	30(11.2)
N/A	1(0.4)
Ways Clinical simulation used in teaching Clinical Skills	
Clinical examination (OSCE)	188(63.1)
Teaching clinical skills	86(28.9)
Learning certain procedures	23(7.7)
Scenario management	1(0.3)
Methods of teaching clinical skills	
Bedside	75(25.2)
Problem-based learning	161(54.0)
Clinical Simulation	62(20.8)

**Table 3:** Perception of the students towards utilization of simulation in clinical teaching

Variable	S.D n(%)	D.A n(%)	N (%)	A	S.A n(%)	Mean	s.d	Decision
[It is incredibly helpful to have someone who works in the field debrief with us after completing a scenario.]	4(1.34)	0(0)	6(2.01)	74(24.83)	214(71.81)	4.65	0.66	S.A
[I find that it’s nice to have simulation experiences, because the nurses are not too helpful, especially during the first few weeks of clinical.]	11(3.69)	30(10.07)	61(20.47)	110(36.91)	86(28.86)	3.75	1.08	A
[After learning on a simulator, instead of saying “i think” I said “i know”. i was confident and i felt like a nurse.]	3(1.01)	14(4.70)	47(15.77)	120(40.27)	114(38.26)	4.10	0.90	A
[Its nice to practice your first-time using simulators without directly working on patients.]	05(1.68)	6(2.01)	10(3.36)	93(31.21)	184(61.74)	4.49	0.80	S.A
[It would be nice to feel more welcomed when coming to use the simulation laboratory]	3(1.01)	4(1.34)	27(9.06)	107(35.91)	107(35.91)	4.37	0.78	S.A
[Its a safe environment to learn and make mistakes in a situation that is fairly realistic]	4(1.34)	6(2.01)	8(2.68)	86(28.86)	194(65.10)	4.54	0.76	S.A
[It helps you recognise clinical abnormalities]	24(8.05)	40(13.42)	45(15.10)	88(29.53)	101(33.89)	3.67	1.28	A
[It increases your awareness in terms of your actual ability and makes you realise your strengths and weaknesses and show you where you need to improve before actually working on a real actual live patient.]	4(1.34)	9(3.02)	15(5.03)	101(33.89)	169(56.71)	4.41	0.82	S.A
[Simulations can help me prepare for clinical placement.]	3(1.01)	10(3.36)	23(7.72)	89(29.87)	173(58.05)	4.40	0.84	S.A
[Simulation helps me get more comfortable with the role of a nurse/doctor/anesthetist]	07(2.35)	9(3.02)	18(6.04)	110(36.91)	154(51.68)	4.32	0.89	S.A
[you don’t really learn many interpersonal skills from practicing on a doll]	30(10.07)	78(26.17)	55(18.46)	67(22.48)	68(22.82)	3.21	1.32	N
[You don’t get a chance to come back to review what you have learnt and try to integrate what you have learnt because of lack of time and accessibility.]	33(11.07)	67(22.48)	64(21.48)	80(26.85)	54(18.12)	3.18	1.27	N
[On a simulator, you can’t tell if you’re actually hurting them. you don’t get the realistic point of view about how the patient really feels.]	15(5.03)	21(7.05)	41(13.76)	106(35.57)	115(38.59)	3.95	1.12	A
[It doesn’t make me that independent to the point where I could do this skill for the first time in a hospital by myself]	25(8.39)	57(19.13)	68(22.82)	98(32.89)	50(16.78)	3.30	1.19	N
[The simulation environment reinforces the importance of organization: although the manikins are not realistic, you can envision yourself being in a hospital setting]	02(0.67)	10(3.36)	32(10.74)	151(50.67)	103(34.56)	4.15	0.79	A
[I think its very stressful and over whelming]	79(26.51)	114(38.26)	4113.76))	45(15.10)	19(6.38)	2.36	1.20	D.A
[Having more variation and unpredictability would make simulations more realistic]	8(2.68)	20(6.71)	61(20.47)	119(39.93)	90(30.20)	3.88	1.00	A
[I am much more confident, because I practiced all the basic skills on simulation, so I am much more prepared for the real-world clinical practice]	21(7.05)	49(16.44)	59(19.80)	98(32.89)	71(23.83)	3.5	1.21	S.A
[It gives you a chance to see things that you won’t see in a clinical setting]	40(13.42)	58(19.46)	53(17.79)	76(25.50)	71(23.83)	3.268456	1.368874	N
[Complex scenarios provide us with amazing opportunities to be able to critically think and apply all that we know.]	4(1.34)	12(4.03)	36(12.08)	116(38.93)	130(43.62)	4.194631	0.8965167	A
[When you are in a simulation laboratory, you are taking it seriously to an extent, but it’s a dummy. you're more careful with a live person]	5(1.68)	23(7.72)	35(11.74)	124(41.61)	111(37.25)	4.05	0.97	A
[It shouldn’t replace clinicals; It should be in addition to it]	10(3.36)	3(1.01)	7(2.35)	66(22.15)	212(71.14)	4.56	0.87	S.A
[We need to manipulate the manikins and feel comfortable with them before we actually begin the scenario to maximize learning time]	8(2.68)	19(6.38)	42(14.09)	122(40.94)	107(35.91)	4.01	0.99	A
[simulations can help students learn how to work in multidisciplinary teams.]	1(0.34)	12(4.03)	24(8.05)	115(38.59)	146(48.99)	4.31	0.81	S.A
[You can’t replace the real world. Nursing students are not having sufficient access to real people; we need that real contact.]	21(7.05)	39(13.09)	56(18.79)	113(37.92)	69(23.15)	3.57	1.18	A
[Faculty could relinquish some control over the equipment. were supposed to be independent learners, yet they don’t trust us to use the equipment in the laboratory.]	15(5.03)	34(11.41)	90(30.20)	69(23.15)	90(30.20)	3.62	1.17	A
[I don’t see the manikins as mimicking a patient. that’s not what am using them for; i am using them to practice my skills.]	16(5.37)	49(16.44)	56(18.79)	100(33.56)	77(25.84)	3.58	1.18	A
[If you are failing it gives you an opportunity to practice further and strengthening your skills.]	4(1.34)	7(2.35)	32(10.74)	105(35.23)	150(50.34)	4.30	0.85	S.A
[We need more academic preparations before using the manikins]	13(4.36)	38(12.75)	62(20.81)	109(36.58)	76(25.50)	3.66	1.12	A
[With the manikins, there's no critical thinking whatsoever; we don’t actually use our brains in anyway. We're just kind of listening]	53(17.79)	96(32.21)	44(14.77)	73(24.50)	32(10.74)	2.78	1.29	N
[It’s a very good opportunity to learn in an environment where they are no risks to a living patient	10(3.36)	20(6.71)	45(15.10)	92(30.87)	131(43.96)	4.05	1.07	A
[You have to get into a certain mind set to get comfortable in the simulation environment]	9(3.02)	30(10.07)	62(20.81)	110(36.91)	87(29.19)	3.79	1.06	A
[It helps to minimize the anxiety when you’re going to practicum. because you know what comes next.]	07(2.35)	14(4.70)	26(8.72)	108(36.24)	143(47.99)	4.22	0.95	S.A
[I think its just making a habit of simulation; we realize that it is a stimulation, but we just don’t make it a reality.]	15(5.03)	38(12.75)	85(28.52)	105(35.23)	55(18.46)	3.49	1.08	A
[With the manikin I can take my time and really feel all the parts.]	16(5.37)	26(8.72)	62(20.81)	84(28.19)	110(36.91)	3.82	1.17	A
**GRAND MEAN**						**3.87**	**0.52**	
**Criterion/Decision mean for 5-point Likert scale**							**3**	

**Table 4:** Satisfaction of the students with use of clinical simulation in teaching and learning

Illuminative Quotes	Initial code	Second order codes	Themes
“ ….I am not satisfied because for the simulations I have had there has been a problem of poor time management. This makes the students who present themselves at the different stations be rushed because they give you little time so that the next group can come and occupy that space so there is that weakness of poor time management[FGD 15b Midwifery]	-poor time management -Space		No of student – space mismatch
“ …I am not satisfied it’s because the number of students assigned to the patient are many and it causes a collision and there is lack of communication ……..” [FGD 13b Midwifery]	-Too many students	-Large number of students	
“………I would say partially it’s ne because simulation is only in 2 departments that is anesthesia and nursing while for medicine we do not do simulation and in nursing where it is the space is actually not enough….” [FGD 3b]	-Space		
“……. I am not satisfied with the level of simulation we are having.… So I feel it would be better in case we have something like theoretically immediately we’ll apply so that we get that knowledge to be impacted onto us, but we already have that so I’m not satisfied…” [FGD A6]	-simulation not aligned with theory classes	Curriculum challenges	Simulation not integrated into the training curriculum
“…I am not satisfied with simulations because we only have them when it is a progressive assessment or there’s someone doing a research study….” [FGD 14b Midwifery]	-Simulation not aligned with normal lectures		Low fidelity of the simulation
“…me I am not satisfied in a way that there is a lot of imagination you will find that you want to administer maybe oxygen they tell you it is already connected like you want to administer any IV fluid you bring a cannular and tie it with a strip so there is a lot of imagination….” [FGD 14b Midwifery]	-A lot of Imagination		
“…..I am not satisfied,,,,yea to shed light on that I can give you like a scenario where you are in an examination and you find out a breach and you know the maneuvers you can do them and you find a fellow student putting on that dummy staff and pushing the baby so in the process of you doing the maneuvers you find that when doing the maneuvers the baby has already come out and the person giving you marks will be like you have done nothing and they fail you from there so…” [FGD 13b]	-simulation not connected to real-world practice		
“… I can say that simulation can equip you with skills but however, when you go to the real life scenario, things are a lot no different…” [FGD A7]	-Different from real world		
“…I find myself being extra confident when I am doing a simulation during simulations because I know at times I am not managing the real life…..” [FGD A6 midwifery]	-not managing the real life		

## Data Availability

Data are available from the corresponding author upon reasonable request and are located in controlled access data https://osf.io/fxnj8/?view_only=e5b397446a304c77b0b7eee4c5eda0f6

## References

[1] Al-Balas M Distance learning in clinical medical education amid COVID-19 pandemic in Jordan: current situation, challenges, and perspectives. 2020. 20: pp. 1–7. 10.1186/s12909-020-02257-4 PMC7530879 33008392

[2] Rassie KJNMJ. The apprenticeship model of clinical medical education: time for structural change. 2017. 130(1461): p. 66–72. 28859068

[3] Johnson JL, Adkins D. and S.J.A.j.o.p.e. Chauvin. Rev Qual Indic rigor qualitative Res 2020. 84(1). 10.5688/ajpe7120 PMC7055404 32292186

[4] Braun V, Clarke V. Using thematic analysis in psychology. Qualitative Res Psychol. 2006;3(2):77–101.

[5] Tong A, Sainsbury P, Craig J. Consolidated criteria for reporting qualitative research (COREQ): a 32-item checklist for interviews and focus groups. Int J Qual Health Care. 2007;19(6):349–57. 17872937 10.1093/intqhc/mzm042

[6] Aalto A-M Social relationships in physicians’ work moderate relationship between workload and wellbeing—9-year follow-up study. 2018. 28(5): p. 798–804. 10.1093/eurpub/ckx232 29365062

[7] Omer TJJoE, Practice. Nursing Students’ Perceptions of Satisfaction and Self-Confidence with Clinical Simulation Experience. 2016. 7(5): pp. 131–138.

[8] Au ML Nursing students’ perception of high-fidelity simulation activity instead of clinical placement: A qualitative study. 2016. 39: p. 16–21. 10.1016/j.nedt.2016.01.015 27006029

[9] Madhavanprabhakaran G, Al-Khasawneh E, Wittmann L.J.S.Q.U.M J. Perceived benefits of pre-clinical simulation-based training on clinical learning outcomes among Omani undergraduate nursing students. 2015. 15(1): p. e105. PMC4318589 25685368

[10] El Naggar MA, Almaeen AH. Students’ perception towards medical-simulation training as a method for clinical teaching. J Pak Med Assoc. 2020;70(4):618–23. 32296205 10.5455/JPMA.6481

[11] García-Mayor S, Nursing students’ perceptions on the use of clinical simulation in psychiatric and mental health nursing by means of objective structured clinical examination (OSCE). Nurse Educ Today. 2021;100:104866. 33735749 10.1016/j.nedt.2021.104866

[12] Codeço A, Assessing clinical simulation as a learning tool when training motivation skills in Periodontology—Students’ perceptions. Eur J Dent Educ. 2020;24(4):644–9. 32396273 10.1111/eje.12544

[13] Elliott H, Brumbaugh K. Student Perceptions of a Simulated Clinical Experience: A Pilot Study. Teaching and Learning in Communication Sciences & Disorders, 2021. 5(1): p. 7.

[14] Alshutwi S, Maintaining clinical training continuity during COVID-19 pandemic: Nursing students’ perceptions about simulation-based learning. Int J Environ Res Public Health. 2022;19(4):2180. 35206368 10.3390/ijerph19042180 PMC8872332

[15] Nel N, E.J.A.J.o.H PE, Stellenberg. Nursing students’ perception of simulation as a clinical teaching method in the Cape Town Metropole. South Afr. 2015;7(2):176–9.

[16] Woodruff K, O’Neill SP, Walton-Moss BJ. Exploring APN students’ perceptions, self-confidence, and satisfaction with clinical simulation. Nurs Educ Perspect. 2017;38(6):347–9. 28570372 10.1097/01.NEP.0000000000000176

[17] García-Mayor S Nursing students’ perceptions on the use of clinical simulation in psychiatric and mental health nursing by means of objective structured clinical examination (OSCE). 2021. 100: p. 104866. 10.1016/j.nedt.2021.104866 33735749

[18] El Naggar MA A.H.J.J.P.M.A., Almaeen. Students’ perception towards medical-simulation training as a method for clinical teaching. 2020. 70(4): pp. 618 – 23. 10.5455/JPMA.6481 32296205

